# Fractionation, identification of chemical constituents, and biological properties of cashew (*Anacardium occidentale* L.) leaf extracts

**DOI:** 10.1002/fsn3.3718

**Published:** 2023-09-23

**Authors:** Dinh‐Chuong Pham, Dieu‐Hien Truong, Quang Huy Tran, Quang Tien Ho, Thieu Anh Duy Nguyen, Thi Ngoc Huyen Nguyen, Thanh Vinh Nguyen, Thi Thao Vy Nguyen, Tan Sang Cao, Colin J. Barrow, Hoang Chinh Nguyen

**Affiliations:** ^1^ Faculty of Applied Sciences Ton Duc Thang University Ho Chi Minh City Vietnam; ^2^ Centre for Sustainable Bioproducts Deakin University Geelong Victoria Australia

**Keywords:** *Anacardium occidentale*, antibacterial, anticancer, antidiabetic, chemical profiling, solvent fractions

## Abstract

The current study aimed to identify the chemical constituents and bioactivities of the crude ethanolic extract (CEE) and its fractions (ethyl acetate (EAF), hexane (HEF), and aqueous (AEF)) from leaves of cashew (*Anacardium occidentale* L.) grown in Vietnam. A total of 31 compounds which belong to alkanes, hydrocarbons, iodine, terpenoids, phenolics, and flavonoids were determined by a gas chromatography–mass spectrometry (GC–MS) analysis, with bis(2‐ethylhexyl) phthalate being the most prevailing compound. The highest total phenolic and flavonoid contents were obtained in the EAF, followed by HEF, CEE, and AQF. All samples showed promising in vitro antibacterial activity, enzyme inhibition, and anticancer activity. Among the samples tested, the EAF exhibited the highest enzyme inhibition activity against α‐amylase and α‐glucosidase (IC_50_ values of 51.24 μg/mL and 99.29 μg/mL, respectively), cytotoxicity activity against HeLa cells (IC_50_ value of 79.49 μg/mL), and antibacterial activity against *Bacillus subtilis* and *Escherichia coli* with MIC values of 5 mg/mL and 2.5 mg/mL, respectively. These findings suggest that the leaves of *A. occidentale* cultivated in Vietnam are a promising source of bioactive components and that EAF is a promising bioactive material warranting further pharmaceutical investigation.

## Graphical abstract

This work aimed to identify the chemical constituents and bioactivities of the *Anacardium occidentale* L. leaf extract and fractions. A total of 31 compounds were determined. The ethyl acetate fraction contained the highest total phenolic and flavonoid contents and consequently showed the highest in vitro antibacterial activity, enzyme inhibition, and anticancer activity, thus warranting further pharmaceutical applications

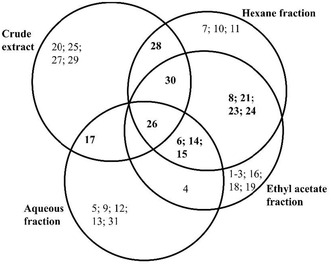

## 1 INTRODUCTION

The overuse of drugs has caused a significant increase in drug resistance. Consequently, the use of natural products has attracted considerable attention to replace synthetic drugs (Do et al., 2020). In recent years, herbal plants have been increasingly used as complementary medicines to prevent or treat various diseases (Baptista et al., 2018; Fabricant & Farnsworth, 2001; Nguyen et al., 2021). Cashew trees (*Anacardium occidentale* L.) have been used in folk medicine worldwide, particularly in America and Africa (Ajileye et al., 2015; Costa et al., 2021; Cruz Reina et al., 2022). A number of bioactive metabolites (e.g., phenolics, flavonoids, anthocyanins, and carotenoids) and biological properties (e.g., antimicrobial, antioxidant, antidiabetic, anticancer, and anti‐inflammatory activities) were found in different parts of cashew trees (da Silva et al., 2022). A sesquiterpene lactone zoapatanolide A was isolated from the ethanol extract of *A. occidentale* cultivated in Nigeria and exhibited potent cytotoxicity against HeLa cells, with an IC_50_ value of 36.2 μM (Taiwo et al., 2017). Cefali et al. (2021) reported the potential use of extracts of cashew cultivated in Brazil for anti‐acne treatment and the prevention of premature skin aging. Costa et al. (2021) noted that the ethanolic extract of the bark of Brazilian cashew trees could be considered an excellent candidate for drug development against cancer and fungal infection. Therefore, *A. occidentale* is considered a potential source of natural bioactive compounds for drug development.

Extraction and separation are two important steps in the isolation of bioactive compounds from organic materials. Different extraction methods, such as Soxhlet extraction, supercritical CO_2_, and maceration, have been used to obtain an extract from different parts of *A. occidentale* (Baptista et al., 2018; Tan et al., 2014). To achieve a bioactive compound‐rich extract and consequently enhance its biological activity, further fractionation or purification steps are required. Among the many separation techniques, liquid–liquid extraction (LLE) can be used to enrich bioactive compounds from an initial extract (Ismail & Chua, 2020; Truong et al., 2021). In this method, the selection of solvents and design of the fractionation process are crucial to obtain the desired compounds. This is because each bioactive compound has a different solubility in different solvents (Truong et al., 2019). Barbosa‐Filho et al. (2015) used solvent partitioning to obtain methanol and ethyl acetate fractions from the ethanol extract of *Anacardium microcarpum* stem bark and these fractions demonstrated potent antibacterial activity against a range of bacteria (*Escherichia coli*, *Pseudomonas aeruginosa*, and *Staphylococcus aureus*). In a previous study, we also successfully obtained a terpenoid‐rich fraction with enhanced biological activities from *Serevenia buxifolia* bark extract (Truong et al., 2021). Therefore, LLE is promising for concentrating different groups of bioactive compounds. Although *A. occidentale* has been studied for its phytochemical constituents and bioactivities (Kossouoh et al., 2008; Osman et al., 2019), the fractionation and identification of specific bioactive components from *A. occidentale* leaves are limited. Particularly, there is no study on the chemical profiles and bioactivities of Vietnamese cashew trees, which are an important economic plant in Vietnam – the largest producer of cashew nuts (Peng et al., 2014).

Considering all the above facts, the aim of the present study was to achieve the bioactive‐rich extract from *A. occidentale* leaves by using LLE. The chemical constituents of the extract and fractions were characterized using gas chromatography–mass spectrometry (GC–MS) analysis. The detailed scientific validation of in vitro antibacterial (*B. subtilis* and *E. coli*), antidiabetic (Type 2 diabetes, α‐glucosidase and α‐amylase) and anticancer (Hela cancer cell line) activities of *A. occidentale* leaf extract and fractions was investigated. The findings of this study could provide new insights into the application of the Vietnamese *A. occidentale* leaf extract in the nutraceutical and pharmaceutical industries.

## 2 MATERIALS AND METHODS

### 2.1 Chemicals

Streptomycin, penicillin G, 3,5‐dinitro salicylic acid (DNS), 4‐nitrophenyl β‐D‐glucopyranoside (pNPG), gallic acid, α‐tocopherol, α‐amylase, α‐glucosidase, acarbose, fetal bovine serum (FBS), l‐glutamine, amphotericin B, and quercetin were obtained from Sigma‐Aldrich. HeLa cells were provided by the American Type Culture Collection. All chemicals were of analytical grade.

### 2.2 Plant material

The mature leaves of *A. occidentale* L. (red cashew tree) were collected from Dong Nai province, Vietnam. The taxonomical identification of the plant was conducted by the VNM Herbarium, Institute of Tropical Biology, Vietnam. The leaves of *A. occidentale* were collected during the flowering–fruiting stage. The leaves were cut, washed carefully with water to remove debris, dried at 45°C for 48 h, and then ground to a powder. The sample was subsequently macerated with 80% ethanol for 24 h at 40°C and the extraction was repeated three times. After extraction and filtration, the ethanol was completely evaporated using a rotary vacuum evaporator, and the sample was subsequently freeze‐dried to obtain the crude ethanolic extract (CEE). The CEE was kept in the dark at 4°C for further experiments.

### 2.3 Fractionation of crude extract by LLE

**FIGURE 1:**
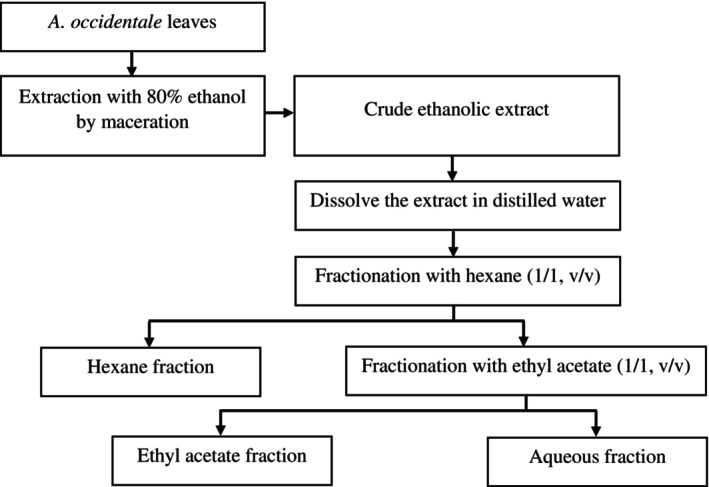
Fractionation of the crude ethanolic extract of *A. occidentale* leaves by liquid–liquid extraction.

The *A. occidentale* leaf extract was fractionated by LLE (Figure 1). Briefly, the CEE was dissolved in distilled water (1:10, w/v), transferred into a separating funnel, equilibrated, and successively fractionated with n‐hexane and ethyl acetate to yield different fractions, respectively called hexane fraction (HEF), ethyl acetate fraction (EAF). The residue was generated, namely aqueous fraction (AQF). The resulting fractions (HEF, EAF, and AQF) were then concentrated using a rotary evaporator. All fractions were then freeze‐dried and kept in the dark at 4°C for further experiments. The fractionation process was repeated three times.

### 2.4 Determination of total flavonoid content and total phenolic content

The total phenolic content (TPC) of the *A. occidentale* leaf extract and its fractions was measured using a modified Folin–Ciocalteu method (Encarnação et al., 2016) with some minor modifications. Briefly, 1 mL of the extract was mixed with 7 mL of diluted Folin–Ciocalteu reagent (1:10, v/v, in water) and 2 mL of 7.5% sodium carbonate. The reaction mixture was then incubated at room temperature for 1 h before measuring the absorbance at 765 nm using a spectrometer (V‐730 UV–Vis Spectrophotometer, Jasco). Gallic acid (50–250 μg/mL) was used to establish a standard curve (*y =* 0.003*x –* 0.0134; *R* ^*2*^ = .9982) for the calculation of the TPC. The TPC was expressed as milligrams of gallic acid equivalents per gram of dry matter (mg GAE/g DM).

The total flavonoid content (TFC) of the crude extract and its fractions was estimated using a modified spectrophotometric method (Medina‐Medrano et al., 2019). Briefly, the extracts (2 mL) were mixed with 5% AlCl_3_ (0.5 mL) and 1 M potassium acetate solution (0.5 mL). The mixture was then incubated at room temperature for 15 min before measuring the absorbance at 415 nm using a V‐730 UV–Vis spectrophotometer (Jasco). Quercetin (20–100 μg/mL) was used as a standard to establish a calibration curve (*y =* 0.01*x +* 0.017; *R* ^*2*^ = .9967) for the calculation of the TFC. The TFC was shown as milligrams of quercetin equivalents per gram of dry matter (mg QE/g DM).

### 2.5 Phytoconstituents identified by GC–MS

The phytoconstituents were analyzed using a GC/MS system (Agilent 7890B/7000C) based on the method of Haron et al. (2019), with minor changes. The dried *A. occidentale* crude extract and its fractions were dissolved in acetonitrile (Sigma) (1:20, w/v). The samples were filtered using a 0.45 μm PTFE filter (Millipore, Sigma‐Aldrich) before injection into GC–MS. The separation was performed with an Agilent DB‐5MS capillary column (40 m × 250 μm × 0.25 μm). The injector was set at 325°C (split mode with the ratio adjusted to 25:1 and an injection volume of 1 μL). The oven temperature started at 70°C for 1 min, increased to 280°C, and was then held for 5 min at 15°C ramp/min. The flow rate of the carrier gas (helium) was maintained at 2.25 mL/min. The mass spectrometer was operated with ionization in the electron impact mode using ionization energy (70 eV) to obtain the mass spectra in the range from 40 to 700 m/z. The chemical profiles were identified based on retention times and by examination of their main spectra against the spectral databases of Wiley and the National Institute Standard and Technology (NIST) MS 2.0. The proportion of each detected component was calculated as the ratio between their area and the total area of all detected compositions from each *A. occidentale* extract or fractions.

### 2.6 Antibacterial activity

#### 2.6.1 Sensitivity test

The bacteria strains (*B. subtilis* ATCC 8012 and *E. coli* ATCC 25922) were collected from stock of culture collections, maintained on trypticase soy agar (TSA), and sub‐cultured monthly. The appropriate bacteria strain was prepared according to the Clinical and Laboratory Standards Institute (CLSI) recommendation, where the OD_600_ value was adjusted to the equivalent of 10^8^ colony‐forming units (CFU)/mL.

The disc diffusion method described by Razmavar et al. (2014) was employed to examine the antibacterial capabilities of *A. occidentale* crude extract and fractions against *E. coli* ATCC 25922 and *B. subtilis* ATCC 8012. A serial concentration (20, 40, 80, and 100 mg/mL) of the crude extract and fractions (HEF, EAF, or AQF) was prepared in 10% of dimethylsulfoxide (DMSO). Subsequently, sterile filter paper disks (6 mm in diameter) were impregnated with 30 μL of each sample (1 mg of tested sample/disk) and allowed to dry at room temperature. Streptomycin (1 mg/mL) and 10% of DMSO were used as the respective standard antibiotic (positive control) and negative control. The tested bacterial suspensions (100 μL) were uniformly spread in petri plates containing TSA medium. The paper disks impregnated with the samples were then placed on the surface of the agar. Finally, the plates were incubated aerobically for 24 h at 37°C, and the antibacterial activity was evaluated by measuring the diameter of the inhibition zone (IZ, mm) (Abew et al., 2014).

#### 2.6.2 Determination of minimum inhibitory concentration and minimum microbicidal concentration values

The minimum inhibitory concentration (MIC) values of the crude extract and fractions were determined using a broth microdilution method with resazurin (Sarker et al., 2007). Resazurin (0.01%) was prepared in sterile distilled water, vortexed well, filter sterilized (0.22 μm filter), and stored at 4°C for further analysis. The crude extract and the fractions were dissolved in DMSO. Serial two‐fold dilutions of the samples (20 mg/mL ‐ 0.078 mg/mL) were then prepared in sterile 96‐well plates containing Mueller–Hinton broth (MHB). Subsequently, 10 μL of bacteria suspension (final concentration of 5 × 10^5^ CFU/mL) and resazurin indicator (10 μL) was added to each well. The plates were then incubated at 37°C for 24 h and the color change was assessed visually. The MIC value was defined as the lowest concentration of the tested samples that prevented the resazurin color change from blue to pink. Each test contained a positive growth (streptomycin) and a negative control (10% DMSO). All experiments were established in triplicate and performed until MICs were constant.

The minimum microbicidal concentration (MMC) value was estimated by plating 10 μL of the test material from the wells where no indicator color change was observed onto the MHB medium. The MMC value was then determined as the lowest concentration without growth (no colony) at the end of the incubation period.

### 2.7 Determination of antidiabetic activity

#### 2.7.1 In vitro α‐amylase inhibition

The α‐amylase inhibition assay was adapted from Thaidi et al. (2020), with minor modifications. Briefly, the samples (crude extract and fractions) were serially diluted in DMSO to obtain different concentrations (25–400 μg/mL). α‐Amylase (3100 U/mg, Sigma) was dissolved in a phosphate buffer solution (pH 6.9) to reach a concentration of 2 U/mL. Soluble starch in water (1%, w/v) was used as the substrate solution. The reaction mixture containing 200 μL α‐amylase solution and 200 μL of each sample was incubated at 37°C for 10 min. Subsequently, 200 μL of the starch solution was added to the reaction mixture and incubated for 10 min at 37°C. The enzyme reaction was then terminated by adding 500 μL of DNS reagent solution (96 mM DNS and 5.31 M sodium potassium tartrate in 2 M NaOH) and placing into boiling water for 10 min. Finally, the mixture was cooled and diluted with 5 mL of distilled water. Acarbose and DMSO were used as the reference standard and control, respectively. The absorbance of the reaction mixture was then measured at 540 nm. The percentage of α‐amylase inhibition (*I*) was estimated as follows:

$$I\;\left(\%\right)=\frac{A_{c}-A_{s}}{A_{c}}\times 100$$

where *A*_c_ and *A*_s_ are the absorbance of the control (DMSO) and the samples, respectively. The results were then shown in terms of the IC_50_ values, which are the concentration of the tested sample required to inhibit 50% enzyme activity.

#### 2.7.2 In vitro α‐glucosidase inhibition

The α‐glucosidase assay was carried out using the method of Damsud et al. (2013), with some changes. The sample (100 μL) and 100 μL of α‐glucosidase (1.0 U/mL) in DMSO were incubated at 25°C for 15 min in the dark before adding 100 μL of 10 mM pNPG solution in DMSO. The reaction solution was then incubated at 25°C for 30 min before adding 300 μL of Na_2_CO_3_ (100 mM). The absorbance was measured at 405 nm using a spectrophotometer. The percentage of α‐glucosidase inhibition was estimated in the same manner as in the α‐amylase assay. Acarbose was also used as a standard (positive control).

### 2.8 Preliminary in vitro cytotoxicity screening

#### 2.8.1 Cell line and culture conditions

HeLa cells were maintained in Eagle's Minimal Essential Medium (EMEM) supplemented with 10% (v/v) FBS, 100 IU/mL penicillin G, 100 μg/mL streptomycin, 0.025 μg/mL amphotericin B, 20 mM HEPES, and 2 mM l‐glutamine at 37°C in a humidified incubator with 5% CO_2_.

#### 2.8.2 Sulforhodamine B assay

The antiproliferative activity of the extracts against HeLa cells was evaluated using the Sulforhodamine B (SRB) assay (Haron et al., 2019). Briefly, the cells were prepared at a density of 5000 cells per well in 96‐well plates at 37°C with 5% CO_2_. The cells were then treated with various concentrations of tested samples (1–100 μg/mL) for 24, 48, and 72 h. Treated cells were fixed with 50% cold trichloroacetic acid (50 μL) for 1 h at room temperature and then washed gently with tap water (5 times) and dried. The cells were subsequently stained with 100 μL of 0.2% (w/v) SRB in 1% acetic acid for 20 min. After five washes with 1% acetic acid, the plate was dried, added with 100 μL of 10 mM Tris base solution, and shaken for 5 min. Finally, the relative cell viability was determined by measuring the absorbance at 540 nm on a 96‐well microtiter plate reader (Synergy HT, Biotek Instruments). Camptothecin (Calbiochem) (1 μg/mL) was used as a positive control. The cytotoxicity was determined as follows (Nguyen et al., 2016):

$$\text{Cytotoxicity}\;\left(\%\right)=\left(1-\frac{{\mathrm{OD}}_{t}}{{\mathrm{OD}}_{c}}\right)\times 100$$

where OD_t_ and OD_c_ are the absorbances of the tested sample and the untreated sample, respectively. The concentration of the sample that is able to inhibit cell proliferation by 50% (IC_50_) was estimated from the dose–response curve.

### 2.9 Statistical analysis

Data were shown as the mean ± standard deviation (SD). Analysis of variance (ANOVA) (Minitab 16 software) and Tukey's multiple comparison test (*p* values < .05) were used to analyze the data.

## 3 RESULTS

### 3.1 TPC and TFC of *A. occidentale* leaf extract and fractions

In this study, the LLE was applied to yield three fractions, namely HEF, EAF, and AQF, from the CEE of *A. occidentale* leaves (Figure 1). The TFC and TPC of the crude extract and its fractions were examined and the results are illustrated in Table 1. Significant variations in the TFC and TPC of the *A. occidentale* crude extract and its fractions were observed in this study (*p* < .05; Table 1). The TFC and TPC showed the following order: EAF > HEF > CEE > AQF. The highest TPC and TFC were obtained in the EAF (233.47 mg GAE/g DM and 43.97 mg QE/g DM, respectively), whereas the recovery of phenolic and flavonoid components was lowest in the AQF (79.47 mg GAE/g DM and 2.0 mg QE/g DM, respectively).

**TABLE 1:** TPC and TFC of the *A. occidentale* leaf extract and fractions.

Extract	TPC (mg GAE/g DM)	TFC (mg QE/g DM)
Crude extract	105.68^c^ ± 0.53	3.73^c^ ± 0.15
Hexane fraction	138.58^b^ ± 1.51	12.30^b^ ± 0.87
Ethylacetate fraction	233.47^a^ ± 0.67	43.97^a^ ± 0.15
Aqueous fraction	79.47^d^ ± 1.70	2.00^d^ ± 0.20

*Note*: Data are the mean ± SD (*n* = 3). Values with different superscript lowercase letters (a–d) within a column are significantly different (*p* < .05).

### 3.2 Phytoconstituents of *A. occidentale* leaf extract and fractions

Table 2 shows the constituents identified from GC–MS chromatography analyses of *A. occidentale* extract and its fractions. A total of 31 chemical components belonging to alkanes, hydrocarbons, iodine, terpenoids, phenolics, and flavonoids were found in the CEE, HEF, EAF, and AQF of the *A. occidentale* leaves (Figure S1).

**TABLE 2:** Phytochemical constituents identified in different *A. occidentale* leaf extract and fractions.

S. No.	CAS number	Name of the compound	Molecular weight (amu)	Retention time (RT, min)	Chemical family
1	062016–14‐2	Octane, 2,5,6‐trimethyl‐	156.188	5.79	Hydrocarbon
2	000544–76‐3	Hexadecane	226.266	5.968	Hydrocarbon
3	007045–71‐8	Undecane, 2‐methyl‐	170.203	6.282	Hydrocarbon
4	000107–50‐6	Cycloheptasiloxane, tetradecamethyl‐	518.132	7.705	Hydrocarbon
5	073105–67‐6	1‐Iodo‐2‐methylundecane	296.1	7.882	Iodoalkane
6	000096–76‐4	Phenol, 2,4‐bis(1,1‐dimethylethyl)‐	206.167	8.019	Phenolics
7	007225–67‐4	Heptane, 2,2,3,3,5,6,6‐heptamethyl‐	198.235	8.031	Alkanes
8	017312–55‐9	Decane, 3,8‐dimethyl‐	170.203	8.299	Alkanes
9	000629–78‐7	Heptadecane	240.282	8.3	Alkanes
10	015356–74‐8	2(4H)‐Benzofuranone, 5,6,7,7a‐tetrahydro‐4,4,7a‐trimethyl‐	180.115	8.511	Benzofuran
11	007212–44‐4	1,6,10‐Dodecatrien‐3‐ol, 3,7,11‐trimethyl‐	222.198	8.677	Terpenoids
12	017312–57‐1	Dodecane, 3‐methyl‐	184.219	10.563	Alkanes
13	002882–96‐4	Pentadecane, 3‐methyl‐	226.266	10.706	Alkanes
14	006418–41‐3	Tridecane, 3‐methyl‐	198.235	11.26	Alkanes
15	000629–94‐7	Heneicosane	296.344	11.266	Aliphatic hydrocarbon
16	014167–59‐0	Tetratriacontane	478.548	12.38	Aliphatic hydrocarbon
17	000629–59‐4	Tetradecane	198.235	13.792	Aliphatic hydrocarbon
18	000630–04‐6	Hentriacontane	436.501	19.273	Aliphatic hydrocarbon
19	000629–99‐2	Pentacosane	352.407	21.41	Aliphatic hydrocarbon
20	000112–95‐8	Eicosane	282.329	22.313	Aliphatic hydrocarbon
21	000638–66‐4	Oxirane, hexadecyl‐	268.277	22.856	Alkanes
22	021964–51‐2	1,15‐Hexadecadiene	222.235	22.868	Alkanes
23	055045–10‐8	Tridecane, 6‐propyl‐	226.266	23.925	Alkanes
24	000301–00‐8	9,12,15‐Octadecatrienoic acid, methyl ester, (Z,Z,Z)‐	292.24	25.205	Alkanes
25	027554–26‐3	1,2‐Benzenedicarboxylic acid, diisooctyl ester	390.277	36.75	Phthalate ester
26	000117–81‐7	Bis(2‐ethylhexyl) phthalate	390.277	40.917	Phthalate ester
27	124201–86‐1	1‐Pyrrolidinebutanoic acid, 2‐[(1,1‐dimethylethoxy)carbonyl]‐.alpha.‐nitro‐, 2,6‐bis(1,1‐dimethylethyl)‐4‐methoxyphenyl ester	520.315	44.3	Phenolics
28	010191–41‐0	Vitamin E (α‐tocopherol)	430.381	47.072	Tocopherols
29	000471–68‐1	Olean‐12‐ene	410.391	49.398	Terpenoids
30	000545–47‐1	Lupeol	426.386	50.421	Terpenoids
31	031897–93‐5	n‐Methyl‐1‐adamantane acetamide	207.162	50.793	Amides

Abbreviations: CAS, chemical abstract service, S. No., serial number.

**FIGURE 2:**
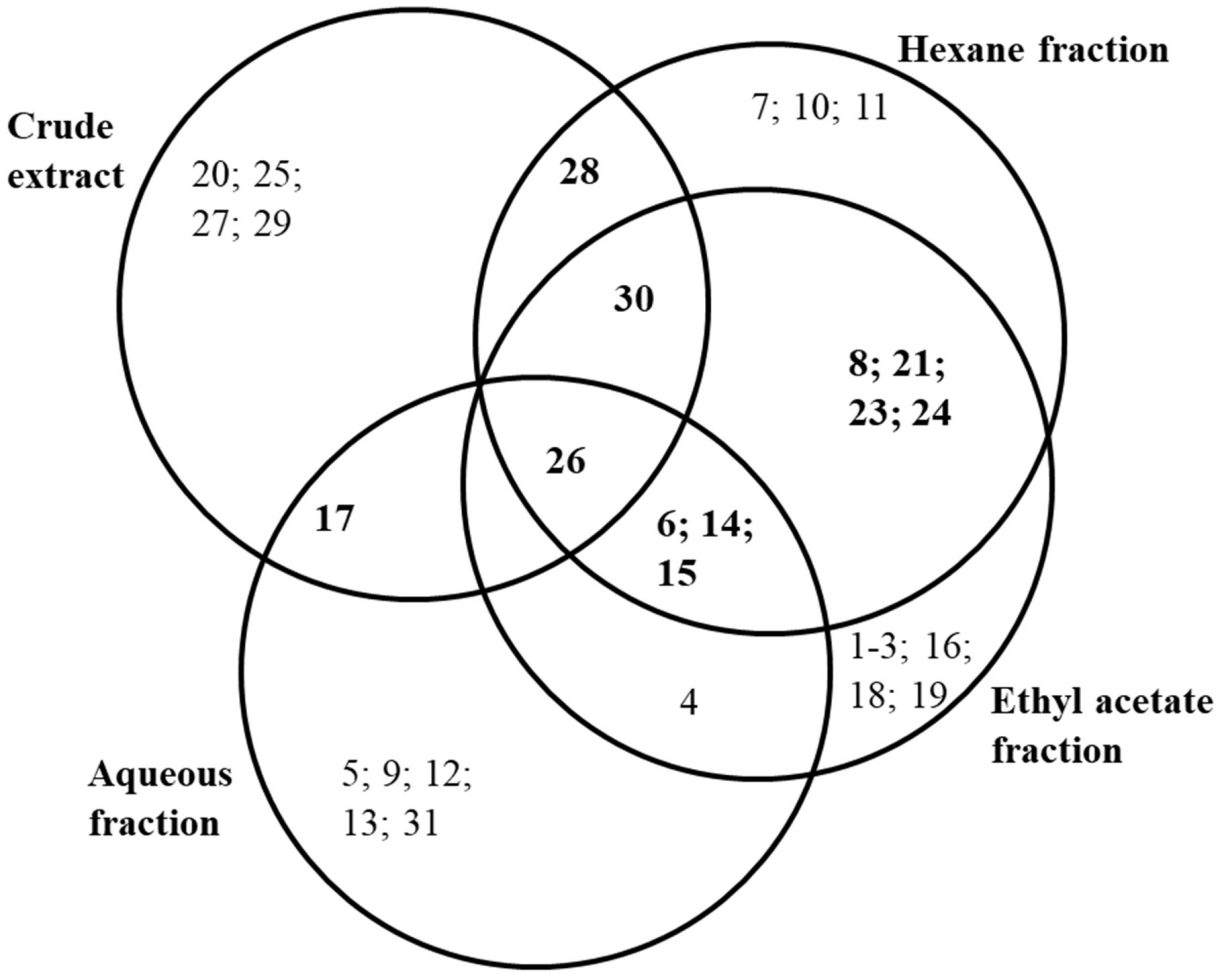
Venn diagram of chemical constituents in the crude ethanolic extract and different fractions of *A. occidentale* leaves. The numbers correspond to the phytoconstituents identified by GC–MS and their names are shown in Table 2.

Eight chemical components were identified from the CEE, representing 25.81% of the phytochemicals identified from the total extract and fractions (Figure 2 and Table S1). The abundant chemical components were found to be bis(2‐ethylhexyl) phthalate (71.90%); 1‐pyrrolidinebutanoic acid, 2‐[(1,1‐dimethylethoxy)carbonyl]‐alpha‐nitro‐, 2,6‐bis(1,1‐dimethylethyl)‐4‐methoxyphenyl ester (5.99%); vitamin E (5.73%); and tetradecane (5.21%), while 1,2‐benzenedicarboxylic acid, diisooctyl ester (BADE) (4.26%); eicosane (3.82%); olean‐12‐ene (3.10%); and lupeol (2.85%) were obtained as minor components (Table 2).

The HEF obtained from the *A. occidentale* leaf CEE led to the identification of 12 organic compounds, accounting for 38.71% of identified compounds in the total extract and fractions (Figure 2; Table S1). The predominant phytoconstituents identified in the HEF were bis(2‐ethylhexyl) phthalate (66.26%), vitamin E (9.42%), and lupeol (5.19%), while the minor chemical components were 2(4 h)‐benzofuranone, 5,6,7,7a‐tetrahydro‐4,4,7a‐trimethyl‐ (4.70%); tridecane, 3‐methyl‐ (3.93%); decane, 3,8‐dimethyl‐ (3.48%); heneicosane (2.48%); tridecane, 6‐propyl‐ (2.19%); 9,12,15‐octadecatrienoic acid, methyl ester, (z,z,z)‐ (2.18%); 1,6,10‐dodecatrien‐3‐ol, 3,7,11‐trimethyl‐ (2.11%); oxirane, hexadecyl‐ (2.27%); and heptane, 2,2,3,3,5,6,6‐heptamethyl‐ (2.05%) (Table 2).

The EAF prepared from the *A. occidentale* leaf extract and analyzed using the same GC–MS conditions led to the identification of 15 chemical constituents, representing 48.39% of phytochemicals in the total extract and fractions (Figure 2; Table S1). The major bioactive compound in the EAF was bis(2‐ethylhexyl) phthalate (72.06%). Other phytoconstituents, such as lupeol (4.06%); tridecane, 3‐methyl‐ (3.37%); decane, 3,8‐dimethyl‐ (3.01%); pentadecane (2.26%); cycloheptasiloxane, tetradecamethyl‐ (2.03%); hentriacontane (1.70%); decane, 3,8‐dimethyl‐ (1.61%); oxirane, hexadecyl‐ (1.56%); phenol, 2,4‐bis(1,1‐dimethylethyl)‐ (1.50%); pentacosane (1.48%); heptadecane, 9‐octyl‐ (1.43%); tetratriacontane (1.37%); octane, 2,5,6‐trimethyl‐ (1.35%); and undecane, 2‐methyl‐ (1.21%), were found as minor components in the EAF (Table 2).

Finally, 12 organic components, which account for 38.71% of identified components in the total extract and fractions from the leaf sample, were found in the AQF (Figure 2; Table S1). The most abundant phytoconstituent was bis(2‐ethylhexyl) phthalate (79.65%), whereas heptadecane (4.18%); tridecane, 3‐methyl‐ (3.66%); cycloheptasiloxane, tetradecamethyl‐ (2.33%); phenol, 2,4‐bis(1,1‐dimethylethyl)‐ (2.24%); 1,15‐hexadecadiene (1.80%); dodecane, 3‐methyl‐ (1.19%); n‐methyl‐1‐adamantaneacetamide (1.17%); 1‐iodo‐2‐methylundecane (1.01%); pentadecane, 3‐methyl‐ (0.96%); heneicosane (0.91%); and tetradecane (0.89%) were found as minor components (Table 2).

### 3.3 Antibacterial activity

This study evaluated the antibacterial activity of the extracts against 1 Gram‐positive (*B. subtilis* ATCC 8012) and 1 Gram‐negative (*E. coli* ATCC 25922) bacteria. Table 3 show the potential antibacterial activity of the tested samples. The crude extract and fractions of *A. occidentale* leaves had different degrees of antibacterial effect. In the sensitivity test, the antibacterial activity of the crude extract and fractions increased (4–14 mm of IZ) with increasing sample concentrations (20–80 mg/mL). At a given concentration, all tested samples showed comparatively less activity against the Gram‐negative (*E. coli*) bacteria than the Gram‐positive (*B. subtilis*) bacteria. Among the samples examined (CEE, HEF, EAF, and AQF), the EAF exhibited the greatest inhibitory effects against two tested bacteria, followed by HEF, CEE, and AQF (Table 3, Figures S2 and S3).

**TABLE 3:** In vitro antibacterial activity of the *A. occidentale* leaf extract and fractions.

Sample	Organisms	Concentrations (mg/mL)	Diameter of zone of inhibition (ZI, mm)^a^
Ethanolic extract	*Escherichia coli* ATCC 25922	20	4.00 ± 1.00
		40	7.00 ± 0.05
		80	9.67 ± 0.58
	*MIC value*	*20*	
	*MMC value*	*>20*	
	*Bacillus subtilis* ATCC 8012	20	6.67 ± 0.58
		40	7.67 ± 0.78
		80	10.67 ± 1.15
	*MIC value*	*>10*	
	*MMC value*	*>10*	
Hexane fraction	*Escherichia coli* ATCC 25922	20	7.33 ± 0.58
		40	9.67 ± 0.58
		80	11.33 ± 0.68
	*MIC value*	*5*	
	*MMC value*	*10*	
	*Bacillus subtilis* ATCC 8012	20	5.33 ± 0.53
		40	8.67 ± 0.23
		80	11.33 ± 0.58
	*MIC value*	*10*	
	*MMC value*	*>10*	
Ethyl acetate fraction	*Escherichia coli* ATCC 25922	20	8.67 ± 0.48
		40	11.67 ± 0.58
		80	13.00 ± 0.02
	*MIC value*	*2.5*	
	*MMC value*	*5*	
	*Bacillus subtilis* ATCC 8012	20	7.00 ± 1.00
		40	10.33 ± 1.15
		80	14.00 ± 1.00
	*MIC value*	*5*	
	*MMC value*	*10*	
Aqueous fraction	*Escherichia coli* ATCC 25922	20	2.00 ± 0.03
		40	3.33 ± 0.45
		80	4.67 ± 0.68
	*Bacillus subtilis* ATCC 8012	20	6.33 ± 0.58
		40	8.00 ± 1.00
		80	9.33 ± 0.78
Streptomycin	*Escherichia coli* ATCC 25922		
	*MIC value*	*0.125*	
	*MBC value*	*0.125*	
	*Bacillus subtilis* ATCC 8012		
	*MIC value*	*0.25*	
	*MBC value*	*0.25*	

^a^ The diameter of ZI of streptomycin (1 mg/mL) for *E. coli* ATCC 25922 and *Bacillus subtilis* ATCC 8012 was 20 mm and 17 mm, respectively. No ZI was observed in the negative control (10% DMSO).

The resazurin reduction test is recommended to examine the antibacterial potential of natural extracts and to determine the MIC and MMC values (Sarker et al., 2007). This method was therefore applied in the present study to estimate the MIC and MMC values of the crude extract and fractions of *A. occidentale* leaves. In this work, the fractions possess better inhibitory effects against the tested bacteria, *E. coli* and *B. subtilis*, than the crude extract. Among the fractions examined, the EAF showed the highest inhibitory activity with MIC values of 2.5 mg/mL against *E. coli* and 5 mg/mL against *B. subtilis*, and MMC values of 5 mg/mL and 10 mg/mL against *E. coli* and *B. subtilis*, respectively. The MIC values of the CEE and AQF were 20 mg/mL against *E. coli* and >10 mg/mL against *B. subtilis*, while the MMC values of these samples were > 20 mg/mL against *E. coli* and *>* 10 mg/mL against *B. subtilis*. The MIC of the HEF was observed to be 5 mg/mL against *E. coli* and 10 mg/mL against *B. subtilis*, while the MMC of this fraction was 10 mg/mL against *E. coli* and >10 mg/mL against *B. subtilis* (Table 3).

### 3.4 Antidiabetic activity

α‐Glucosidase and α‐amylase play an important role in the management of hyperglycemia‐linked diabetic disease (Tiwari, 2015). In this work, the ability of the crude extract and different fractions from *A. occidentale* leaves to inhibit α‐glucosidase and α‐amylase was examined, and the results are presented in Table 4. All the tested extracts (CEE, HEF, EAF, and AQF) inhibited α‐amylase and α‐glucosidase activity in a concentration‐dependent manner (25–400 μg/mL). At a concentration of 400 μg/mL, the EAF showed the highest inhibitory activity against α‐amylase (75.84%) and α‐glucosidase (71.19%) while the HEF had a moderate inhibition of α‐amylase (61.80%) and α‐glucosidase (60.68%). In contrast, the CEF and AQF showed no significant difference in the inhibitory activity against α‐amylase (57.17% and 57.18%, respectively) and α‐glucosidase (54.04% and 53.88%, respectively).

**TABLE 4:** α‐glucosidase and α‐amylase inhibitory activities of the crude ethanolic extract and its fractions from *A. occidentale* leaves.

Activities	Concentration (μg/mL)	Inhibition (%)				
		CEE	HEF	EAF	AQF	Acarbose
α‐Amylase inhibition	25	11.47^d^ ± 0.19	29.34^c^ ± 0.40	38.58^b^ ± 3.19	10.52^d^ ± 0.19	47.85^a^ ± 1.92
	50	24.30^d^ ± 2.31	32.54^c^ ± 0.59	46.90^b^ ± 3.18	22.24^d^ ± 0.69	56.12^a^ ± 1.74
	100	35.78^c^ ± 3.88	39.24^c^ ± 1.00	55.80^b^ ± 1.14	36.18^c^ ± 1.26	65.52^a^ ± 1.13
	200	42.40^d^ ± 0.90	53.34^c^ ± 1.00	64.44^b^ ± 3.53	43.28^d^ ± 1.39	71.82^a^ ± 1.12
	400	57.17^d^ ± 2.19	61.80^c^ ± 1.13	75.84^b^ ± 1.29	57.18^d^ ± 1.26	96.70^a^ ± 1.15
	IC_50_	304.15^a^ ± 1.25	198.26^b^ ± 2.56	51.24^c^ ± 1.30	302.91^a^ ± 1.56	48.13^d^ ± 1.12
α‐Glucosidase inhibition	25	11.09^d^ ± 0.66	21.80^c^ ± 0.24	29.51^b^ ± 1.45	9.87^d^ ± 0.57	33.73^a^ ± 1.23
	50	21.57^d^ ± 0.96	30.50^c^ ± 1.14	40.37^b^ ± 1.01	20.61^d^ ± 1.30	42.94^a^ ± 1.06
	100	33.52^c^ ± 1.07	38.61^c^ ± 0.57	50.79^b^ ± 2.18	37.82^c^ ± 4.06	58.78^a^ ± 1.06
	200	41.38^d^ ± 1.35	51.76^c^ ± 1.17	62.55^b^ ± 1.95	43.20^d^ ± 1.52	65.54^a^ ± 1.17
	400	54.04^d^ ± 1.70	60.68^c^ ± 1.52	71.19^b^ ± 1.11	53.88^d^ ± 2.13	94.20^a^ ± 1.17
	IC_50_	313.98^a^ ± 2.54	193.35^b^ ± 1.24	99.29^c^ ± 2.34	311.73^a^ ± 2.45	90.77^d^ ± 2.56

The inhibitory effect of the extracts on α‐glucosidase and α‐amylase were also assessed using the IC_50_ values. Among the tested samples, the EAF revealed the highest inhibitory capacity against α‐amylase and α‐glucosidase with IC_50_ values of 51.24 μg/mL and 99.29 μg/mL, respectively. These values were slightly higher than those of a standard antidiabetic drug, acarbose (IC_50_ values of 48.13 μg/mL and 90.77 μg/mL against α‐amylase and α‐glucosidase, respectively), indicating that EAF is a promising antidiabetic agent. However, the IC_50_ values for α‐glucosidase and α‐amylase inhibition by other tested samples were significantly higher than that of EAF and acarbose (Table 4).

### 3.5 Preliminary cytotoxicity effect

**FIGURE 3:**
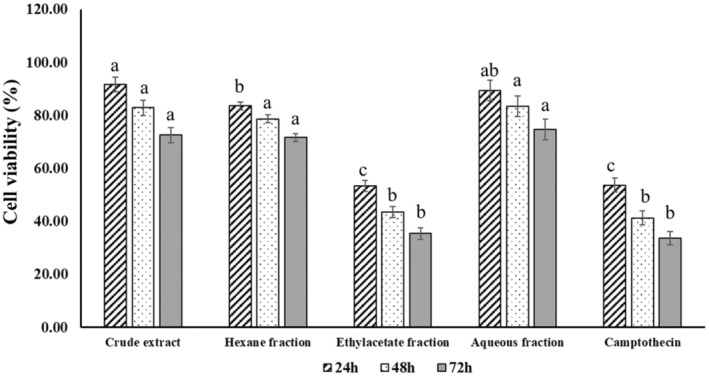
The cytotoxicity of the crude ethanolic extract and fractions (100 μg/mL) from *A. occidentale* leaves at different treatment times (24, 48, and 72 h).

Figure 3 presents the viability of HeLa cells treated with different extracts (CEE, HEF, EAF, and AQF) at a concentration of 100 μg/mL and an anticancer drug (camptothecin). All tested samples exhibited significant antiproliferative activity against HeLa cells in a time‐dependent manner (*p* < .05). Among the samples tested, EAF was the most active fraction, resulting in the lowest viability of HeLa cells of 53.56%, 43.61%, and 35.54% for 24, 48, and 72 h of treatment, respectively. Notably, its antiproliferative activity against HeLa cells was comparable to camptothecin (no significant difference at all treatment times), indicating that EAF is a promising anticancer agent for further application. However, the HEF, AQF, and CEE showed significantly lower activity than the EAF. Hence, treatment of HeLa cells for 48 h with the EAF was chosen for further analysis.

**FIGURE 4:**
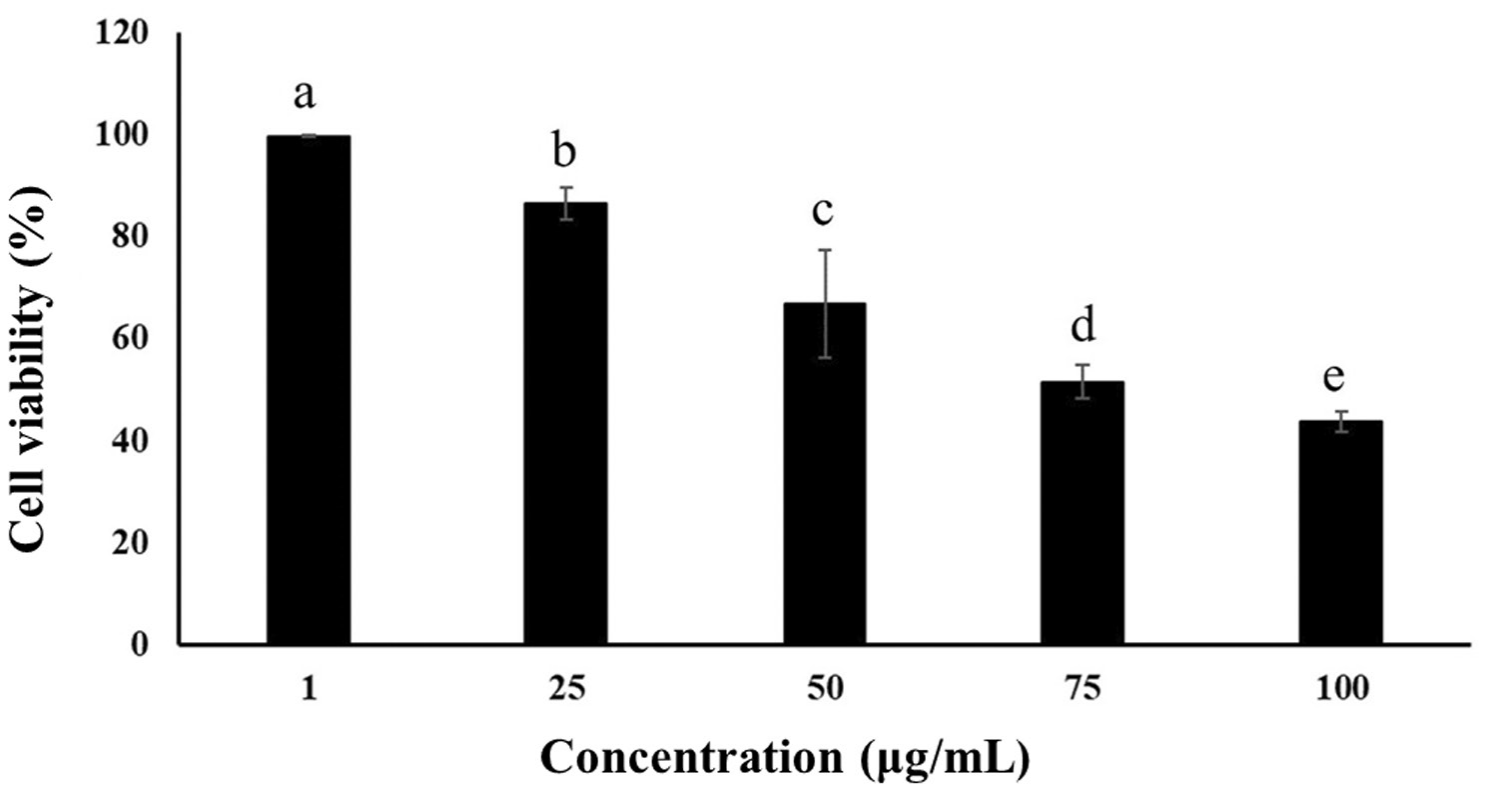
Viability of HeLa cells treated with different concentrations of ethyl acetate fraction (1–100 μg/mL). Different letters indicate statistical significance at *p* < .05.

The viability of the HeLa cells also changed in a concentration‐dependent manner (Figure 4). Increasing the EAF concentration led to a significantly decreased viability of HeLa cells after 48 h of incubation (*p* < .05). The EAF showed the most potent activity against HeLa cells with an IC_50_ of 79.49 μg/mL, whereas the IC_50_ values of the other tested extracts were > 100 μg/mL.

## 4 DISCUSSION

LLE is an established method to achieve the separation of compounds with different polarities (e.g., phenolics and flavonoids) (Alara et al., 2021). In this work, LLE was applied to fractionate the ethanolic extract of *A. occidentale* leaves using different solvents. Consequently, HEF, EAF, and AQF were obtained from the CEE. These extracts revealed the presence of different phytoconstituents, including alkanes, hydrocarbons, iodine, terpenoids, phenolics, and flavonoids. Phenolics and flavonoids are known as important bioactive compounds in cashew plants and exhibit potent bioactivities (Salehi et al., 2020). Therefore, the content of these compounds in the crude extract and its fractions (HEF, EAF, and AQF) was evaluated. The results revealed that the TPC and TFC showed statistically significant differences among the extract and fractions (*p* < .05). The EAF showed the highest TPC (233.47 mg GAE/g DM) and TFC (43.97 mg QE/g DM), followed by the HEF, CEE, and AQF. This could be due to the chemical components of the phenolic and flavonoid groups being more semi‐polar to non‐polar. Consequently, these compounds were present at high levels in the EAF. The solvent is an important factor affecting the success of extraction and fractionation (Nur et al., 2019). Solvent selection is based on the principle of “like dissolves like,” where a component can dissolve in a solvent with similar polarity (Amaro et al., 2015). The findings obtained in this work are similar to that of Nur et al. (2019) who obtained the highest content of phenolics and flavonoids in the ethyl acetate fraction from the crude extract of paku atai tuber.

The biological activities of an extract are attributed to its bioactive constituents and their concentration. In this study, EAF showed the highest antibacterial activity against two tested bacteria (*B. subtilis* and *E. coli*), inhibitory capacity against α‐amylase and α‐glucosidase, and cytotoxicity against HeLa cells. This could be because the EAF contained the highest level of phenolics and flavonoids. These compounds are known to possess potent bioactivities (Salehi et al., 2020). To provide more information about the chemical constituents and biological activities of *A. occidentale* extracts and fractions, GC–MS analysis was applied and showed the presence of 31 phytochemical compounds, which most likely contribute to the bioactivities of the extracts (Salehi et al., 2020; Sassi et al., 2022). The highest number of components (15) were found in the EAF, followed by the HEF (12), AQF (12), and CEE (8) (Table S1). Therefore, the highest antibacterial, antidiabetic, and anticancer activities of the EAF could be correlated to the occurrence of more bioactive components in this fraction. Cell death of a cancerous tumor due to effect of EAF from herbal plants was reported in the study of Idris et al., 2017. Cancer cells could be dying by secondary necrotic under the induction of EAF after 24 h of treatment. The release of secondary necrotic is advantageous to allow the efficient activation of the immune system that relates to the clearance of tumor cells from the tumor microenvironment. Besides, cells may also be died by apoptosis at earlier time‐post‐treatment and switch to secondary necrosis over time (Idris et al., 2017; Vakkila & Lotze, 2004).

Among the bioactive compounds identified, bis(2‐ethylhexyl) phthalate (di‐(2‐ethylhexyl) phthalate (DEHP)), a member of the flavonoid family, was the most abundant compound in all extracts (71.90%, 60.26%, 72.06%, and 79.65% in the CEE, HEF, EAF, and AQF, respectively) (Table S1). Huang et al. (2021) revealed that DEHP is a natural phthalic acid ester (e.g., in the essential oils produced by *Clerodendrum inerme, Ziziphus mauritiana, Pyrus ussriensis*, and *Cirsium japonicum*). This compound isolated from *Calotropis gigantean* flowers exerts potent antibacterial activity against *B. subtilis* with a MIC value of 32 μg/mL (Habib & Karim, 2009).

Lupeol, a natural triterpenoid, was identified in the chemical profiles of three extracts: HEF (5.19%), EAF (4.06%), and CEE (2.85%). This compound is widely found in the extracts of fruits, vegetables, and medicinal plants (Liu et al., 2021; Saleem, 2009) and exhibits various pharmaceutical effects including antioxidant, anti‐inflammatory, antimicrobial, and anticancer activities (Cmoch et al., 2008; Liu et al., 2021; Somwong & Theanphong, 2021). Cmoch et al. (2008) revealed that lupeol could induce the death of cancer cells, such as cervical carcinoma HeLa (IC_50_ = 37 μM), breast carcinoma MCF‐7 (IC_50_ = 50 μM), and lung carcinoma Ạ‐549 (IC_50_ = 50 μM). Recently, Somwong and Theanphong (2021) detected the presence of lupeol in ethanolic extracts of *Derris scandens*, *Albizia procera*, and *Diospyros rhodocalyx* plants, and the potent anti‐inflammatory activity of these extracts correlated to the content of lupeol.

Two bioactive compounds, namely tridecane, 3‐methyl‐ and heneicosane, were detected in the chemical profiles of three fractions (HEF, EAF, and AQF), but not in the CEE. Heneicosane, an aliphatic hydrocarbon, exhibited excellent antimicrobial and antioxidant activity (Rhetso et al., 2020; Vanitha et al., 2020). The ethyl acetate extract of *Lumbago zeylanica* leaves containing heneicosane showed potent antimicrobial activity against *Aspergillus fumigatus* (IZ = 29 mm) and *Streptococcus pneumoniae* (ZI = 31 mm) at a concentration of 10 μg/mL (Vanitha et al., 2020). In addition, other bioactive metabolites identified from the extract and fractions of *A. occidentale* leaves (e.g., vitamin E; tetradecane; decane, 3,8‐dimethyl‐; oxirane, hexadecyl‐; tridecane, 6‐propyl‐; eicosane; BADE; 1‐pyrrolidinebutanoic acid, 2‐[(1,1‐dimethylethoxy)carbonyl]‐alpha‐nitro‐, 2,6‐bis(1,1‐dimethylethyl)‐4‐methoxyphenyl ester; olean‐12‐ene; heptane, 2,2,3,3,5,6,6‐heptamethyl‐; 2(4 h)‐benzofuranone, 5,6,7,7a‐tetrahydro‐4,4,7a‐trimethyl‐; 1,6,10‐dodecatrien‐3‐ol, 3,7,11‐trimethyl‐; and 9,12,15‐octadecatrienoic acid, methyl ester, (z,z,z)‐, etc) could contribute to the biological activities of the samples tested (Naeim et al., 2020; Okechukwu, 2020; Rhetso et al., 2020; Shah et al., 2022; Yadav et al., 2018). These compounds isolated from other sources have been reported to possess potent pharmacological effects such as antibacterial, anticancer, antioxidant, antidiabetic, and anti‐inflammatory activities (Chan et al., 2016; Devi & Muthu, 2014; Khan & Javaid, 2022; Tleubayeva et al., 2022; Viswanathan & Sheeba Gnanadeebam, 2015). These findings indicated that the leaves of *A. occidentale* cultivated in Vietnam could be a promising source of bioactive compounds and the *A. occidentale* leaf extract, particularly EAF, could be used as a promising bioactive agent for the treatment of bacterial infection, diabetes, or human cervical cancer.

## 5 CONCLUSION

This work reports the extraction and fractionation of bioactive compounds from *A. occidentale* leaves. An ethyl acetate fraction contained the highest level of phenolics and flavonoids (233.47 mg GAE/g DM and 43.97 mg QE/g DM, respectively) and exhibited the highest inhibitory activity against *B. subtilis* and *E. coli*, α‐amylase and α‐glucosidase, and showed cytotoxicity against HeLa cells. A total of 31 bioactive compounds were identified and potentially responsible for the observed bioactivities of the extracts. This study suggests that the ethyl acetate fraction from *A. occidentale* leaf extract or its constituents has potential as leads for pharmaceutical applications.

## AUTHOR CONTRIBUTIONS

**Dinh‐Chuong Pham:** Conceptualization (equal); formal analysis (equal); investigation (equal). **Dieu‐Hien Truong:** Conceptualization (equal); methodology (equal); project administration (equal); resources (equal); supervision (equal); writing – original draft (equal). **Quang Huy Tran:** Investigation (equal); validation (equal). **Quang Tien Ho:** Formal analysis (equal); investigation (equal). **Thieu Anh Duy Nguyen:** Data curation (equal); formal analysis (equal). **Thi Ngoc Huyen Nguyen:** Validation (equal); visualization (equal). **Thanh Vinh Nguyen:** Validation (equal); visualization (equal). **Thi Thao Vy Nguyen:** Data curation (equal); validation (equal). **Tan Sang Cao:** Validation (equal); visualization (equal). **Colin J. Barrow:** Conceptualization (equal); supervision (equal); writing – review and editing (equal). **Hoang Chinh Nguyen:** Conceptualization (equal); methodology (equal); project administration (equal); resources (equal); supervision (equal); writing – review and editing (equal).

## CONFLICT OF INTEREST STATEMENT

The authors have declared no conflicts of interest for this article.

## ETHICS STATEMENT

Ethical approval is not applicable in this paper.

## Supporting information


Appendix S1

## Data Availability

The data presented in this study are available on request from the corresponding author.
