# Supplementary material for: Fractionation, identification of chemical constituents, and biological properties of cashew (Anacardium occidentale L.) leaf extracts
Source: Food Sci Nutr. 2023 Sep 23;11(12):7996–8008. doi: 10.1002/fsn3.3718 (PMC10724627; doi:10.1002/fsn3.3718)
Supplement: Supplementary file 1 — Appendix S1 [file FSN3-11-7996-s001.docx]

**Supplementary Material**


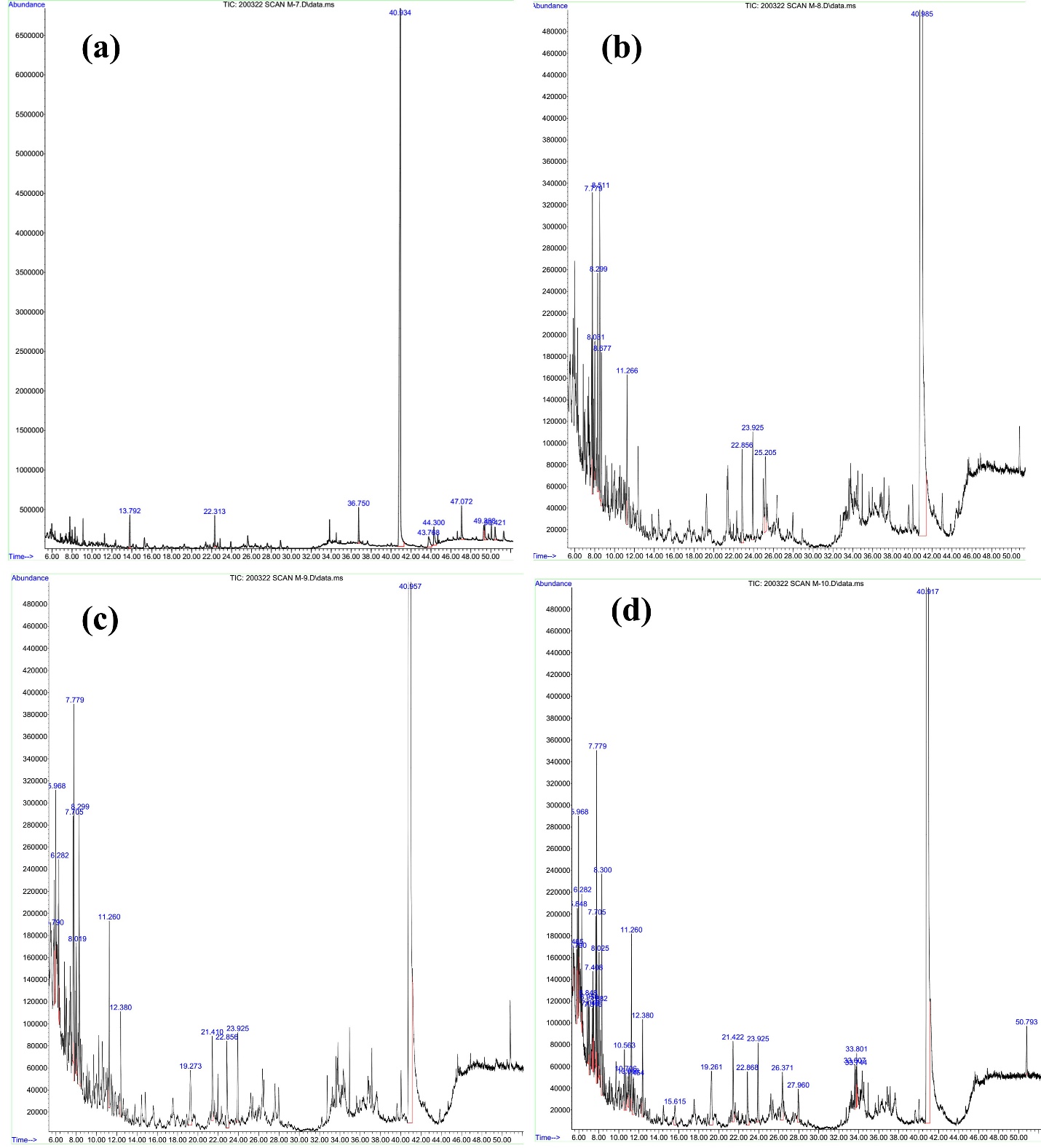


**FIGURE S1** Gas chromatography-mass spectrometry (GC-MS) chromatograph of phytoconstituents in the crude ethanolic extract (a), hexane fraction (b), ethyl acetate fraction (c), and aqueous fraction (d) from *A. occidentale* leaves.


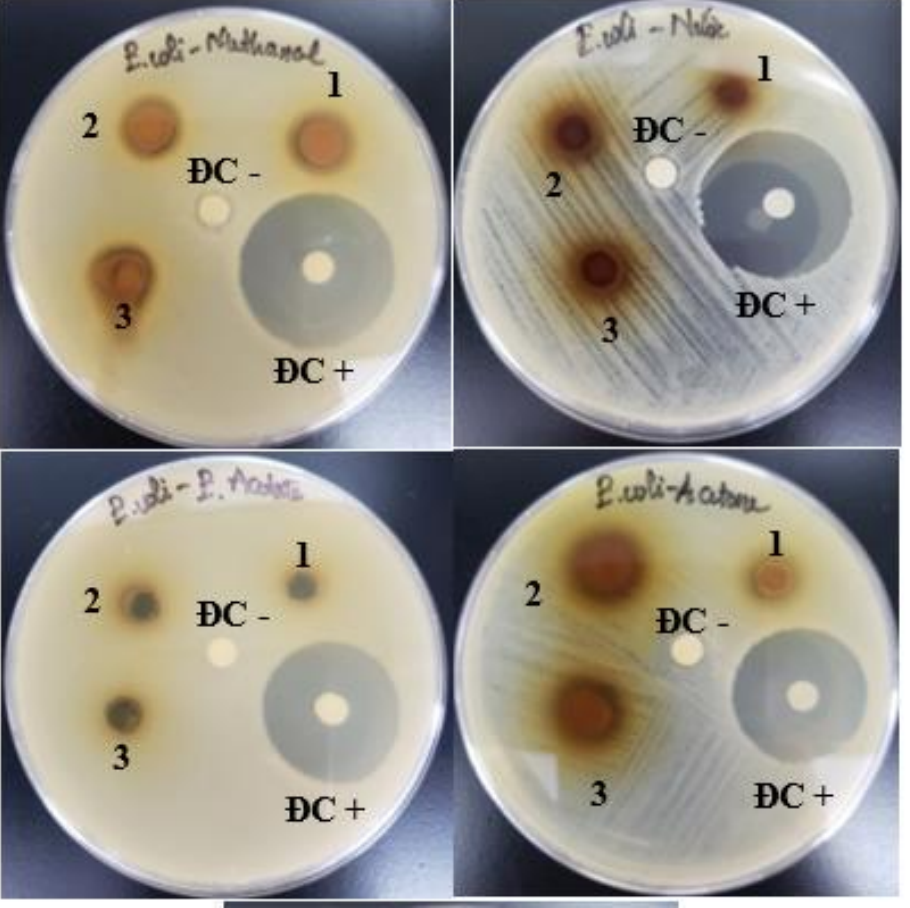


**D**

**C**

**A**

**B**

**FIGURE S2** *In vitro* anti-*Escherichia coli* activity of the *A. occidentale* leaf extract and fractions. A, ethanolic extract; B, hexane fraction; C, aqueous fraction; and D, ethyl acetate fraction. 1, 2 and 3 correspond to 20, 40 and 80 mg/mL respectively. ĐC -, negative control; ĐC +, positive control.


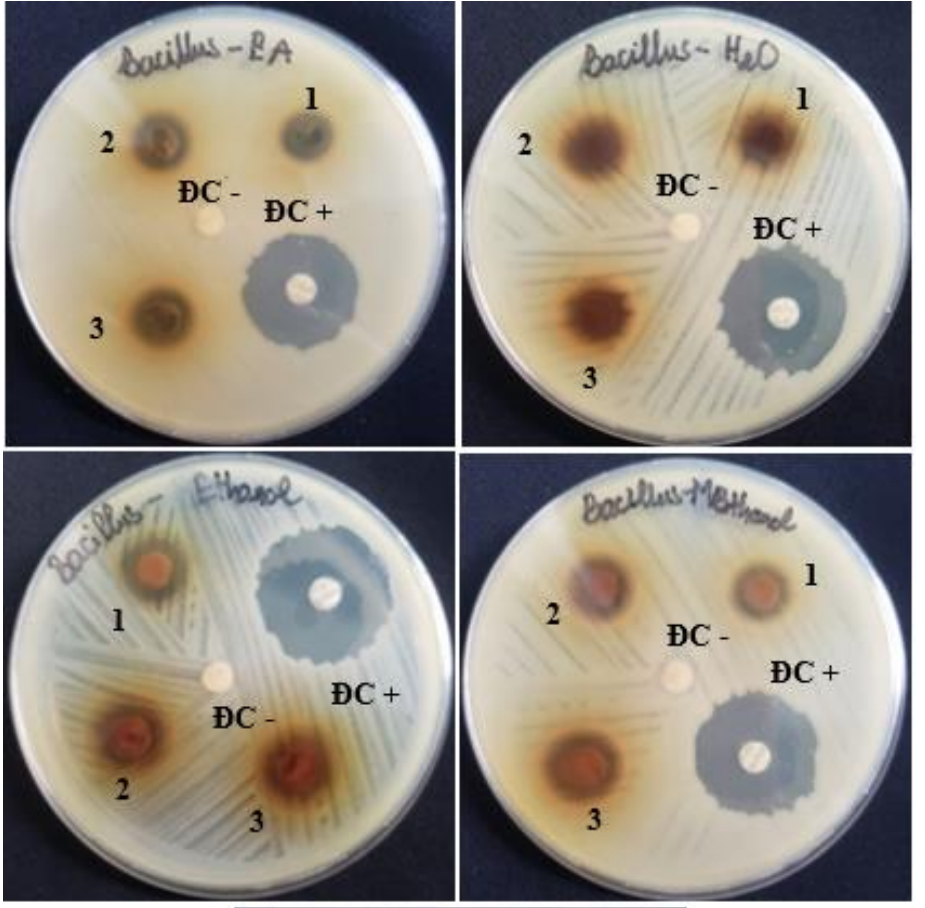


**D**

**C**

**B**

**A**

**FIGURE S3** *In vitro* anti-*Bacillus subtilis* activity of the *A. occidentale* leaf extract and fractions. A, ethanolic extract; B, aqueous fraction; C, ethyl acetate fraction; and D, hexane fraction. 1, 2 and 3 correspond to 20, 40 and 80 mg/mL respectively. ĐC -, negative control; ĐC +, positive control.

**TABLE S1** GC–MS analysis for the crude extract and fraction from *A. occidentale* leaves

| **S. No.** | **Samples** | **CAS number** | **Name of the compound** | **Molecular weight (amu)** | **Retention time (RT, min)** | **Peak area (%)** |
| --- | --- | --- | --- | --- | --- | --- |
|  | **Crude extract** | | | | | |
| 1 |  | 000629-59-4 | Tetradecane | 198.235 | 13.792 | 5.21 |
| 2 |  | 000112-95-8 | Eicosane | 282.329 | 22.313 | 3.82 |
| 3 |  | 027554-26-3 | 1,2-Benzenedicarboxylic acid, diisooctyl ester | 390.277 | 36.75 | 4.26 |
| 4 |  | 000117-81-7 | Bis(2-ethylhexyl) phthalate | 390.277 | 40.934 | 71.90 |
| 5 |  | 124201-86-1 | 1-Pyrrolidinebutanoic acid, 2-[(1,1-dimethylethoxy)carbonyl]-.alpha.-nitro-, 2,6-bis(1,1-dimethylethyl)-4-methoxyphenyl ester | 520.315 | 44.3 | 5.99 |
| 6 |  | 010191-41-0 | Vitamin E (α-tocopherol) | 430.381 | 47.072 | 5.73 |
| 7 |  | 000471-68-1 | Olean-12-ene | 410.391 | 49.398 | 3.10 |
| 8 |  | 000545-47-1 | Lupeol | 426.386 | 50.421 | 2.85 |
|  | **Hexane fraction** | | | | | |
| 9 |  | 006418-41-3 | Tridecane, 3-methyl- | 198.235 | 7.779 | 3.93 |
| 10 |  | 007225-67-4 | Heptane, 2,2,3,3,5,6,6-heptamethyl- | 198.235 | 8.031 | 2.02 |
| 11 |  | 017312-55-9 | Decane, 3,8-dimethyl- | 170.203 | 8.299 | 3.48 |
| 12 |  | 015356-74-8 | 2(4H)-Benzofuranone, 5,6,7,7a-tetrahydro-4,4,7a-trimethyl- | 180.115 | 8.511 | 4.70 |
| 13 |  | 007212-44-4 | 1,6,10-Dodecatrien-3-ol, 3,7,11-trimethyl- | 222.198 | 8.677 | 2.11 |
| 14 |  | 000629-94-7 | Heneicosane | 296.344 | 11.266 | 2.48 |
| 15 |  | 000638-66-4 | Oxirane, hexadecyl- | 268.277 | 22.856 | 2.05 |
| 16 |  | 055045-10-8 | Tridecane, 6-propyl- | 226.266 | 23.925 | 2.19 |
| 17 |  | 000301-00-8 | 9,12,15-Octadecatrienoic acid, methyl ester, (Z,Z,Z)- | 292.24 | 25.205 | 2.18 |
| 18 |  | 000117-81-7 | Bis(2-ethylhexyl) phthalate | 390.277 | 40.985 | 60.26 |
| 19 |  | 010191-41-0 | Vitamin E (α-tocopherol) | 430.381 | 47.072 | 9.42 |
| 20 |  | 000545-47-1 | Lupeol | 426.386 | 50.421 | 5.19 |
|  | **Ethylacetate fraction** | | | | | |
| 21 |  | 062016-14-2 | Octane, 2,5,6-trimethyl- | 156.188 | 5.79 | 1.35 |
| 22 |  | 000544-76-3 | Hexadecane | 226.266 | 5.968 | 1.61 |
| 23 |  | 007045-71-8 | Undecane, 2-methyl- | 170.203 | 6.282 | 1.21 |
| 24 |  | 000107-50-6 | Cycloheptasiloxane, tetradecamethyl- | 518.132 | 7.705 | 2.03 |
| 25 |  | 006418-41-3 | Tridecane, 3-methyl- | 198.235 | 7.779 | 3.37 |
| 26 |  | 000096-76-4 | Phenol, 2,4-bis(1,1-dimethylethyl)- | 206.167 | 8.019 | 1.50 |
| 27 |  | 017312-55-9 | Decane, 3,8-dimethyl- | 170.203 | 8.299 | 3.01 |
| 28 |  | 000629-62-9 | Heneicosane | 212.25 | 11.266 | 2.26 |
| 29 |  | 014167-59-0 | Tetratriacontane | 478.548 | 12.38 | 1.37 |
| 30 |  | 000630-04-6 | Hentriacontane | 436.501 | 19.273 | 1.70 |
| 31 |  | 000629-99-2 | Pentacosane | 352.407 | 21.41 | 1.48 |
| 32 |  | 007390-81-0 | Oxirane, hexadecyl- | 268.277 | 22.856 | 1.56 |
| 33 |  | 055045-10-8 | Tridecane, 6-propyl- | 226.266 | 23.925 | 1.43 |
| 34 |  | 000117-81-7 | Bis(2-ethylhexyl) phthalate | 390.277 | 40.957 | 72.06 |
| 35 |  | 000545-47-1 | Lupeol | 426.386 | 50.421 | 4.06 |
|  | **Aqueous fraction** | | | | | |
| 36 |  | 000629-59-4 | Tetradecane | 198.235 | 6.848 | 0.89 |
| 37 |  | 000112-95-8 | Cycloheptasiloxane, tetradecamethyl- | 282.329 | 7.705 | 2.33 |
| 38 |  | 073105-67-6 | 1-Iodo-2-methylundecane | 296.1 | 7.882 | 1.01 |
| 39 |  | 000096-76-4 | Phenol, 2,4-bis(1,1-dimethylethyl)- | 206.167 | 8.025 | 2.24 |
| 40 |  | 000629-78-7 | Heptadecane | 240.282 | 8.3 | 4.18 |
| 41 |  | 017312-57-1 | Dodecane, 3-methyl- | 184.219 | 10.563 | 1.19 |
| 42 |  | 002882-96-4 | Pentadecane, 3-methyl- | 226.266 | 10.706 | 0.96 |
| 43 |  | 006418-41-3 | Tridecane, 3-methyl- | 198.235 | 11.26 | 3.66 |
| 44 |  | 000629-94-7 | Heneicosane | 296.344 | 15.615 | 0.91 |
| 45 |  | 021964-51-2 | 1,15-Hexadecadiene | 222.235 | 22.868 | 1.80 |
| 46 |  | 000117-81-7 | Bis(2-ethylhexyl) phthalate | 390.277 | 40.917 | 79.65 |
| 47 |  | 031897-93-5 | N-Methyl-1-adamantane acetamide | 207.162 | 50.793 | 1.17 |
